# President’s Address to the Michigan Dental Association

**Published:** 1900-07-15

**Authors:** H. C. Raymond


### PRESIDENT'S ADDRESS TO THE MICHIGAN DENTAL ASSOCIATION.

BY H. C. RAYMOND, D D S.

*Mr Chairman, and Members of the Michigan Dental Association:*—I tried to get out of making an address, but as that was impossible, I have made it just like myself as abbreviated as possible.

Probably there is no truer saying than the one, "There is nothing new under the sun" It is being demonstrated continually in all its phases in our walks and vocations through life; and nowhere is it better or more frequently brought to mind than in our own profession, as may be seen in almost every issue of the dental journals, where one and another man springs up claiming priority for certain inventions, or introductions of new ideas, which they claim they practiced, or saw practiced, or introduced, or discussed long ago. There is really nothing new; and when one prides himself on being truly original he is oftener than not only giving expression to ideas coincidental in another man's brain or re-introducing methods in another form, many of which were far from being either new or original many generations ago.

Now, this introduction or prologue is simply a means of letting myself down easy, in case you should not be disposed to do so, for my not giving you something entirely new in the President's address to the Association.

I am not attempting to give anything new, for the simple reason that I have nothing new to give, owing to my head being still pretty full of what I should term unfinished business of the profession. One sometimes gets tired of old subjects and questions, but many of them can not be put away, and some will not be put away but are continually before us.

You know it is the steady hammering and continual dredging that brings about what we are aiming for, so I am going in as few words as possible to hammer at and dredge out one or two subjects which, while they are not by any means new to us, are far from being fully shaped or cleared away to the liking of many of us.

I hope to see at this meeting a larger gathering than at any of our past State meetings not only of members but of those who are not members. I also hope that the greater number of those who are not members will become members before this meeting closes. Why is it that, with some seven hundred dentists in the State, there are only some one hundred and fifty members of this Association? And why do so few of the members take active interest in the meetings, or, as, in some cases, even neglect to support the Association by paying the dues?

Do the members of the profession forget that it is due to societies and associations such as this, that we *are* a profession to-day; that we have our dental colleges, and that the wonderful advancements have been made which marks the dental profession as a leader over all other professions in the progress achieved during the last few decades? It may be that our Code of Ethics so often alluded to is a stumbling block to many joining. But why? While it may be, and probably is in some respects objectionable, it is really only a binder or means of strengthening us and keeping us together, as a Mason's oaths and secrets are the stronghold of the order.

If, as has been advanced, the Code of Ethics was made for the protection of its members against flagrant advertisers and quacks, how could its purpose be better effected than by every man who has any claims at all to be called professional joining the Association, and thus form a means of so educating the public in dental matters and teaching them the difference between the truly professional and competent man and the charlatan?

Such an Association, strong in numbers and largely represented in every corner of the State, each and every member doing his share in enlightening the public and aided by the Association as a body in the work, would make it impossible for quackery to exist.

I will not go further on the question of educating the public in dental matters, as we are to have a paper during this meeting on that subject, and if I allow myself to warm up too much I might possibly steal some of the essayist's thunder, and so cause some of the strong points in his paper to fall flat. But as a means to enlarging the membership of the State Association and to increase the interest in Association work, I would make a strong plea for the formation of dental societies in the smaller towns—in some cases two or more adjacent towns might form joint societies. This would keep up an active interest in such work and act as a feeder to the mother society, besides keeping the dentists in friendly touch with each other and forming the good fellowship between them that ought always to exist.

Dr. Neal, in the *Cosmos* of last January, has an article which is well worth careful reading, in which he speaks among other things of the benefits accruing from membership in our societies, and of what a commercial or advertising dentist loses, and of the anomolous position he holds in life. But time will not permit me to quote from it. This question of a large membership in our State Associations would also go far in helping to adjust the existing condition of affairs relative to State Examining Boards, and with the knowledge of the manner in which the affairs of some of the State Examining Boards throughout the country have been and are being conducted, notably in the States of California and Wisconsin. I think it will be admitted that radical action is required in that connection. It is very doubtful if State Examining Boards should be allowed unlimited power at any rate, unless the appointment of the members on the Board be controlled or influenced by the State Association. One of the first Examining Boards of New York was an Examining Board only, without any power to grant licenses. It was upon their recommendation that the State Society, of which the examiners were practically an integral part, granted diplomas to successful candidates. Though there have been some changes, the examiners are still chosen by the State Society; they are, therefore, always under the supervision of that society, and their unblemished record is a powerful argument against a system which allows any politics to enter into and govern the appointments, a system which now unfortunately prevails in so many States. From the—I can only call it rotten—condition existing in some of the State Examining Boards and the character of some of the men forming them, I think the time is ripe for a decided limitation of the powers vested in State Dental Examiners. I would like to see a State law that would restrict the duties of the Examining Boards to examinations only, and the licensing power to the State Associations; or at least for the State Society to so control the appointments on the Board that the examiners making it up might be men of such character and integrity that the power of granting licenses would be perfectly safe in their hands. I would like to see at this meeting a resolution from its members carrying with it such a recommendation and forwarded to the Chief Executive of the State, though not necessarily to the present Governor.

What does a Governor of a State and his adherents know about making such appointments for the best interest of the public? The men who are the best political wire pullers and who will take off their coats and work for the party in power are the men who get the jobs, irrespective of any fitness they may possess.

Who are more capable of knowing what is best for the community in this respect than the members of this Association, especially if the membership be large, as I have indicated it might, and could be?

Politics should no more enter into the appointments of State Dental Examiners than they should enter into the election of officers in this or any other society of its kind. It is simply a question of putting men in such positions who are educated and in every way fitted to fill them.

I can not help repeating that it is right here where a strong State Dental Society with a large membership, such as it should have, could control this matter, than which there is none more important, bearing as it does on the right of the advertising charlatans and illegitimate practitioners to gull and fleece the uninformed portion of the public, and to unlawfully compete with those who are properly qualified, and who are working to raise the dental profession to the place it is entitled to and which it will eventually attain.

And now, while a slap has been given the advertising dentist, and as there has been considerable difference of opinion aired lately in regard to colleges advertising, it will not be out of place to say a few words in that connection. When a college advertises, as with a practitioner, of course it is the intent of the advertisement, and not the question of the right of either the institution or the man to advertise. It has been remarked that a door-plate, a card, or even a display of the degree is advertising. There seems, by common consent, to be a certain amount of advertising permissible within the limits of professional dignity, so the question resolves itself into the extent to which a school or a professional man may go. It certainly seems that the rule or restriction, that what is good for a man after he leaves college is good for the college while he is attending it; and it does not appear to be a hard question as to how far the school may go in advertising their clinics and still live up to the spirit of the Code of Ethics Is it likely the student will have higher ideals than his teachers? Where are we to look if not to our teachers for professional morals? It is questionable if a clinic should be used as a means of revenue in an educational institution. When it is used as such, it frequently enters into direct competition with the profession in that community. It might be advanced as an argument, and probably not without some reason, that the public understands what the word dentist means, but that many of the class of people from whom the clinics depend upon for their material for operating, etc., have not a full understanding of the meaning of clinic. Probably this may be in a measure true, and if the dental profession would take it upon itself to inform those too poor to employ a regular practitioner of the advantages of the dental clinic it might in a measure help to settle this question, and there would be less reason for colleges to advertise. Some colleges do not in any way advertise their clinics, those in charge rightfully believing that the Code applies fully as much to colleges as to practitioners of dentistry. If some can and do get along without it, why should not all? There is something here for the National Association of Dental Faculties to decide, and decide decisively and promptly.

Some colleges not only advertise their clinics in the public press, giving prominence to the cheapness of the work performed, etc., but insert the names of the men in charge, and thus openly violate at least the spirit of the

Code of Ethics and apparently take advantage of their positions to bring themselves before the public. Is this fair or right?

Dr. Dunbar, in a letter to *Items of Interest* last summer, gets directly to the gist and foundation of the matter when he says: "If it is unethical for a graduate to advertise in the manner quoted (referring to some of the college advertisements) it is certainly more reprehensible in his sponsors, and no amount of sophistical argument would ever convince a right thinking profession to the contrary." If these sound views, and such they certainly are, were followed it would be of infinite benefit to educational institutions, and would effectually settle this very important question.

The schools are members of the Faculties Association; the teachers condemn individuals for the practices they themselves are guilty of. Should the schools be thus favored? I think not But with all these things before us we must not be pessimistic, we have every reason rather to look at the bright side of affairs, for, with many of our vexed questions yet unsettled, the tendency of our profession is still upward, and we have much left to fight for.

One of the most gratifying forward moves is the one recently taken by the dental college of the State University in getting the course increased to four years. We should all be gratified at such a move, for four years is none too long for the dental course. It has been shown that in the three years' course the best students had all and more than they could do in the time, and many of them much more than they could do. The very best rarely pass perfect examinations, and the poorest hardly succeed in passing at all.

The ability of nearly every dentist is sometimes ques tioned. We all make failures, and our course at school is none too long. Then the lengthening of the course, and at the same time raising the standard of the preliminary education will go far in solving the question of the over-production of dentists, and in a measure aid the legitimate practitioner in his competition with quackery; as with a high standard for the preliminary, strictly enforced, it would weed out in the beginning many of the unfit who now enter our colleges, and the longer course would give the student and his teachers better opportunities of finding out his fitness or unfitness for a dental career. This longer course will also graduate men who will be more competent in every way to practice in the learned and scientific manner our profession demands and equip them to be teachers and instructors of those they come in contact with, for such every professional man should be.

Before leaving the college question I would like to say a word in reference to the tendency of some of the dental colleges to draw away from the medical departments and in trying to teach some of the sciences within their own walls which purely belong to the medical section. To me this is a step in the wrong direction, for by leaving that part which is best taught in the medical department to that department, it gives more opportunity for the dental faculty to devote their time to the strictly dental studies Besides, we should keep as close to the medical degree as we can. It would be one of the best moves for the elevation of our profession if a man could take the M.D. degree, and I think it is not one of the impossibilities of the future that this will come about.

Another point and I have finished. The dental department at Ann Arbor is, I believe, a child of this Association, and as such should not be lost sight of the way it seems to have been in the last few years. Our Constitution provides for the appointment of a University Visiting and Consulting Committee, but the clause seems in some way to have become a dead letter as no such committees have been appointed for some time. There should be such a committee, and it should be an active one, and not one in name only, for what could bring better results than the conference of such committees with those in power at the college, being composed as it ought to be of some of the graduates of the University of Michigan, and having all its best interests at heart

Gentlemen, you will no doubt agree with what I said to commence with, viz: that I would advance nothing new. I really had something new up my sleeve, but these old themes have so completely taken possession of me that it has got away, so I will not weary you by trying to find it.

If I have said anything to start you thinking again, or still better, if I succeed in bringing about active measures in relation to one or more of the questions I have re-introduced, I will feel amply repaid for the midnight oil I have consumed in preparing to promulgate other men's views and opinions, and I will rest satisfied that I have not taken up your time to no purpose.

---

On motion of Dr. Fowler, the President's address was referred to a committee of three, which should report at some future session. The President appointed as this committee Drs. Hoff, E. A. Honey and A. Diack.

Owing to the absence of all the members of the Board of Censors, by special motion, the President was instructed to appoint a new Board. Accordingly, Drs. James, Porter and Oakman were appointed.

The Committee on Publication, through Dr. Noble, reported that the DENTAL REGISTER had offered to report and print the proceedings of the meeting and supply each member of the Association with a copy of the DENTAL REGISTER containing the report, without expense to the Association. On motion the report was approved and adopted.

Dr. Honey, for local Committee of Arrangements, then made a report, which was accepted and adopted.

The President then called upon Dr. Porter to read his paper.

